# Multi-Fidelity Local Surrogate Model for Computationally Efficient Microwave Component Design Optimization

**DOI:** 10.3390/s19133023

**Published:** 2019-07-09

**Authors:** Yiran Song, Qingsha S. Cheng, Slawomir Koziel

**Affiliations:** 1 Department of Electrical and Electronic Engineering, Southern University of Science and Technology, Shenzhen 518055, China; 2 School of Electronics and Information Engineering, Harbin Institute of Technology, Harbin 150001, China; 3 School of Science and Engineering, Reykjavik University, Menntavegur 1, IS-101 Reykjavik, Iceland; 4 Faculty of Electronics, Telecommunications and Informatics, Gdansk University of Technology, 80-233 Gdansk, Poland

**Keywords:** local surrogate model, multi-fidelity optimization, space mapping, bandpass microstrip filter, compact UWB antenna, MIMO antenna

## Abstract

In order to minimize the number of evaluations of high-fidelity (“fine”) model in the optimization process, to increase the optimization speed, and to improve optimal solution accuracy, a robust and computational-efficient multi-fidelity local surrogate-model optimization method is proposed. Based on the principle of response surface approximation, the proposed method exploits the multi-fidelity coarse models and polynomial interpolation to construct a series of local surrogate models. In the optimization process, local region modeling and optimization are performed iteratively. A judgment factor is introduced to provide information for local region size update. The last local surrogate model is refined by space mapping techniques to obtain the optimal design with high accuracy. The operation and efficiency of the approach are demonstrated through design of a bandpass filter and a compact ultra-wide-band (UWB) multiple-in multiple-out (MIMO) antenna. The response of the optimized design of the fine model meet the design specification. The proposed method not only has better convergence compared to an existing local surrogate method, but also reduces the computational cost substantially.

## 1. Introduction

Internet of Things (IoT) technology allows a large number of electronic devices interconnected wirelessly [1,2]. It demands higher data transmission rate and higher operating frequency than the current communication technology (e.g., 5G) can offer. The microwave components for IoT applications require performances such as wide frequency bands, high power, and small size. Therefore, the structure of these components have become increasingly complex [3,4,5,6,7]. Reliable and computationally efficient design for high performance microwave components is one of the major challenges in IoT technology.

By encoding given design specifications into an objective function (typically related to S-parameters evaluated over certain frequency bands), the microwave component design task can be formulated as a nonlinear optimization problem, and solving this problem yields a better design. In the complex microwave component optimization problem, high-fidelity electromagnetic (EM) simulation allows accurate response evaluation but its computation is expensive. It is impracticable to use high-fidelity EM simulation models directly for numerical optimization in many cases, because conventional optimization algorithms (e.g., gradient-based methods and global optimization methods) require a large number of response evaluations (EM simulations) to converge.

Currently, the most promising approach to realize computationally efficient optimization of expensive computational models is surrogate-based optimization (SBO) [8,9,10]. It is used as a predictive tool that identifies promising locations in the search space, and then, the new candidate solution obtained is verified with reference to the high-fidelity (or “fine”) model. There are two major types of surrogate models.

The first one is a response surface approximation model constructed from sampled high-fidelity simulation data. A large variety of approximation (and interpolation) techniques are available, including polynomial regression, radial basis functions, Kriging interpolation, and artificial neural networks. If the design space is sampled at sufficient density, the surrogate model created will be a reliable representation of the microwave component of interest so that it can be used for optimization purposes. Unfortunately, the computational overhead of constructing a globally accurate approximation model may be very significant due to data acquisition. Moreover, the number of training samples required to ensure the sufficient accuracy will grow rapidly with the dimensions of the problem.

Physics-based models constitute the other type of surrogate model. They are constructed from underlying low-fidelity (or “coarse”) model of the structure being studied. In general, low-fidelity models can be obtained in the following ways: (1) equivalent circuit models using circuit and microwave theory (e.g., circuit simulation module in ADS); (2) lower fidelity simulation models, in other words, simulation with coarser discretization of the microwave structure. However, the low-fidelity models are typically simulation-based, and in many cases, the computational cost of these models can significantly affect the overall cost of the SBO process.

An important consideration is the trade-off between model accuracy and evaluation speed when choosing simulation-based low-fidelity models as surrogate models. A multi-fidelity EM-based optimization approach is proposed [11]. It exploits a series of coarse-discretization models with different fidelity to perform the sequential optimization process. These models are evaluated by the same EM solver as the fine model. The optimal design of the current model is used as the initial design of the next (higher fidelity) model. At the last step the final design is refined by using an auxiliary response surface model.

In this work, we propose a novel multi-fidelity local surrogate optimization algorithm. A series of local (region) surrogates are constructed iteratively using the sample data of multi-fidelity coarse models and response surface approximations. The size and moving direction of the local region are updated using the current local optimal solution. The last local surrogate is then refined by space mapping techniques to obtain an optimal design for the fine model. The proposed algorithm is demonstrated through design of a bandpass microstrip filter and a compact UWB MIMO antenna. In both cases, the proposed approach is compared to a local surrogate optimization algorithm (“original algorithm”) [12] as well as to direct optimization of the fine model. It can be seen that the proposed approach has great computational savings compared to the direct optimization algorithm. Compared with the original algorithm, the multi-fidelity local surrogate optimization algorithm can find a more satisfactory optimal solution that achieve the design requirements.

## 2. Multi-Fidelity Local Surrogate Model Optimization

In this section, we present the multi-fidelity surrogate model based on local response surface approximations. In the optimization process, local region modeling and optimization are performed iteratively. The judgment factor is introduced to adaptively change local region size, so that the algorithm converges quickly and accurately to the optimal solution. The last surrogate model is refined by space mapping to obtain the optimal design with higher accuracy.

### 2.1. Microwave Component Design Problem

A microwave component design task can be formulated as a nonlinear minimization problem

(1) $$x_{}^{*}\in \arg\;\underset{x}{\min}U\left(R_{f}(x)\right),$$

where $x\in {ℜ}^{n}$ is a vector of *n* design variables The vector $x^{*}$ is the optimal design to be found. $R_{f}\in {ℜ}^{m}$ represents a fine model response vector obtained by EM simulation at high computational costs (e.g., the transmission coefficient $\left|S_{21}\right|$ of a component evaluated at *m* different frequencies). U(·) is a scalar merit function related to a design specification. In many cases, the objective function can be defined in the norm sense as $U\left(R_{f}(x)\right)=\left\|R_{f}(x)-R_{T}^{*}\right\|$, where $R_{T}^{*}$ is a target response (or design specification). It is impractical to directly solve the above problem using conventional optimization techniques (e.g., quasi-Newton algorithm and genetic algorithm, etc.). These techniques usually require a large number of the fine model evaluations, resulting in prohibitive computational cost.

The SBO approach aims at addressing the aforementioned difficulties [13]. It speeds up the design process by transferring the optimization burden to inexpensive yet reasonably accurate surrogate models. Thus, the original design problem (1) is replaced by an iterative procedure

(2) $$x^{\left(i+1\right)}=\arg\;\underset{x}{\min}U\left(R_{s}^{\left(i\right)}(x)\right).$$

The procedure produces a sequence of designs $x^{\left(i\right)},\;i=0,1,\ldots$. Each one is the optimal design for the *i*th surrogate model $R_{s}^{\left(i\right)}$ respectively. Assuming that the accuracy of surrogate models are increasing in optimization process, the design sequence can eventually converge to the optimal design of the fine model.

### 2.2. Multi-Fidelity Local Surrogate Models

The design optimization technique proposed here utilizes a series of coarse models (coarse-discretization EM models) $\{R_{c.j}\},j=1,\;\ldots ,\;K,$ evaluated by the same EM solver as fine model but faster. It is recommended that the number of models *K* is less than 4. The fidelity of these models can be adjusted by controlling the mesh density through model parameters (specific to a given EM solver, e.g., cells per wavelength or edge refinement, etc.). Therefore, the total number of simulated meshes of a component and its computational cost can be reduced. The first coarse model $R_{c.1}$, is the fastest and the least accurate. The last one $R_{c.K}$, is closest to the fine model accuracy. There is an important trade-off between the computational complexity and accuracy of the coarse models, which greatly affects the performance of the surrogate-based optimization process. In particular, it is essential that the coarse model capture all important features present in the fine model (e.g., the general “shape” of responses etc.).

The surrogate models are constructed from simulation data of coarse models at various accuracy using an approximation-based response surface method. This method can be applied to the cases where the computational cost of coarse models cannot be ignored, such as the coarse-discretization EM model of complex microwave devices or antenna structures.

Let $x^{\left(i,j\right)}={\left[x_{1}^{\left(i,j\right)}\;x_{2}^{\left(i,j\right)}\;\ldots \;x_{n}^{\left(i,j\right)}\right]}^{T}$ be a certain design obtained in the *i*th surrogate model optimization built with the *j*th coarse model $R_{c.j}$. $x^{\left(0,1\right)}$ is denoted as the initial design of the 1st surrogate model built using the 1st coarse model $R_{c.1}$. Instead of optimization $R_{c.j}$ directly, the *i*th surrogate model is built using the 2nd-order response surface model in the region containing $x^{\left(i,j\right)}$, defined as $\left[z^{\left(i,j\right)}-d^{\left(i,j\right)},z^{\left(i,j\right)}+d^{\left(i,j\right)}\right]$, where $d^{\left(i,j\right)}={\left[d_{1}^{\left(i,j\right)}\;d_{2}^{\left(i,j\right)}\;\ldots \;d_{n}^{\left(i,j\right)}\right]}^{T}$. Initial region size determined by $d^{\left(0,1\right)}$ usually equal 2% to 10% of $x^{\left(0,1\right)}$, and its region center $z^{\left(0,1\right)}$ coincides with $x^{\left(0,1\right)}$.

We construct the surrogate model using 2*n*+1 sample points of evaluated $R_{c.j}$ at $z^{\left(i,j\right)}$ and at boundary of the region $\left[z^{\left(i,j\right)}-d^{\left(i,j\right)},z^{\left(i,j\right)}+d^{\left(i,j\right)}\right]$. The coordinates of these samples are $z_{k}^{\left(i,j\right)}={\left[z_{1}^{\left(i,j\right)},\ldots ,z_{k}^{\left(i,j\right)}+\mathrm{sign}(k)d_{k}^{\left(i,j\right)},\ldots ,z_{n}^{\left(i,j\right)}\right]}^{T},$ k=−n,−n+1,…,0,…,n−1,n. Then the local surrogate model for the *i*th region is $s^{\left(i,j\right)}(x)={\left[s_{1}^{\left(i,j\right)}\;s_{2}^{\left(i,j\right)}\ldots \;s_{m}^{\left(i,j\right)}\right]}^{T}$, where *m* is the number of frequency points of responses. The *l*th frequency response of the surrogate model $s_{l}^{\left(i,j\right)}(x)$ is defined as

(3) $$s_{l}^{\left(i,j\right)}(x)=s_{l}^{\left(i,j\right)}({\left[x_{1}\cdots x_{n}\right]}^{T})=\lambda_{l.0}^{\left(i,j\right)}+\lambda_{l.1}^{\left(i,j\right)}x_{1}+\cdots +\lambda_{l.n}^{\left(i,j\right)}x_{n}+\lambda_{l.n+1}^{\left(i,j\right)}x_{1}^{2}+\cdots +\lambda_{l.2n}^{\left(i,j\right)}x_{n}^{2},$$

where $\lambda_{l.r}^{\left(i,j\right)},l=1,\ldots ,m,\;r=0,1,\ldots ,2n,$ is the *r*th coefficient of the 2nd-order response surface model. The model coefficients are obtained by solving the liner regression problem

(4) $$\left[\begin{array}{ccccc} 1 & \begin{array}{cc} z_{-n.1}^{\left(i,j\right)} & \cdots \end{array} & z_{-n.n}^{\left(i,j\right)} & \begin{array}{cc} {\left(z_{-n.1}^{\left(i,j\right)}\right)}^{2} & \cdots \end{array} & {\left(z_{-n.n}^{\left(i,j\right)}\right)}^{2}\\ \vdots & \begin{array}{cc} \vdots & \end{array} & \vdots & \begin{array}{cc} \vdots & \end{array} & \vdots \\ 1 & \begin{array}{cc} z_{0.1}^{\left(i,j\right)} & \cdots \end{array} & z_{0.n}^{\left(i,j\right)} & \begin{array}{cc} {\left(z_{0.1}^{\left(i,j\right)}\right)}^{2} & \cdots \end{array} & {\left(z_{0.n}^{\left(i,j\right)}\right)}^{2}\\ \vdots & \begin{array}{cc} \vdots & \end{array} & \vdots & \begin{array}{cc} \vdots & \end{array} & \vdots \\ 1 & \begin{array}{cc} z_{n.1}^{\left(i,j\right)} & \cdots \end{array} & z_{n.n}^{\left(i,j\right)} & \begin{array}{cc} {\left(z_{n.1}^{\left(i,j\right)}\right)}^{2} & \cdots \end{array} & {\left(z_{n.n}^{\left(i,j\right)}\right)}^{2} \end{array}\right]\left[\begin{array}{c} \lambda_{l.0}^{\left(i,j\right)}\\ \lambda_{l.1}^{\left(i,j\right)}\\ \vdots \\ \lambda_{l.2n}^{\left(i,j\right)} \end{array}\right]=\left[\begin{array}{c} R_{-n.l}^{\left(i,j\right)}\\ \vdots \\ R_{0.l}^{\left(i,j\right)}\\ \vdots \\ R_{n.l}^{\left(i,j\right)} \end{array}\right],$$

where $R_{k.l}^{\left(i,j\right)}$ is a *l*th element of the coarse model response vector $R_{c.j}(z_{k}^{\left(i,j\right)})$. $R_{c.j}(\cdot )$ is an *m*-dimensional coarse model frequency response function vector over a specific frequency band (e.g., the return loss |*S*_11_|). Therefore, in building a surrogate, the coefficients are solved by (4) separately at each frequency point.

### 2.3. Surrogate Model Optimization with Adaptive Region Updating

This algorithm is an iterative process of local modeling and optimization. Therefore, the size and moving direction of each local region are two important factors, which affect the accuracy of the optimal design and the convergence speed of the algorithm. First, a local surrogate model $s^{\left(0,1\right)}$ is established using $R_{c.1}$ and optimized in the region containing the initial design similar to [12], until the new local optimal design lies within the interior of the current region. At this time, we regard as the optimal design of the surrogate model at the lowest accuracy is found.

Then, this optimal design is used as the regional center of the next higher-accuracy local surrogate model (related to $R_{c.2}$) and the new surrogate model is established and optimized using the above process. The process of establishing and optimizing the local surrogate model at the same accuracy is defined as one iteration of our algorithm.

The fidelity of the local surrogate increases with the number of iterations. In addition, the current optimal design serves as a starting point for the next iterative process. The size of local region at beginning of each iteration is adaptively updated according to a judgment factor. The judgment factor of the (*i*+1)th surrogate can be expressed as $\delta_{r}^{\left(i+1\right)}$, defined as

(5) $$\delta_{r}^{\left(i+1\right)}=\frac{1}{m}\sum_{l=1}^{m}\beta_{l}r_{l}^{\left(i+1\right)},$$

where $\beta_{l}$ can adjust the weight of the judgment factor at different frequencies if needed (here, $\beta_{l}=1,\;l=1,\;\ldots ,\;m$). $r_{l}^{\left(i+1\right)}$ representing the similarity between the surrogate model and coarse model at the *l*th frequency is defined for,

(6) $$r_{l}^{\left(i+1\right)}=\frac{R_{c.j}^{l}(x^{\left(i+1,j\right)})-R_{c.j}^{l}(x^{\left(i,j\right)})}{s_{l}^{\left(i+1,j\right)}(x^{\left(i+1,j\right)})-s_{l}^{\left(i,j\right)}(x^{\left(i,j\right)})},$$

where $R_{c.j}^{l}$ is the *l*th component of the coarse model response vector $R_{c.j}$.

The judgment factor $\delta_{r}^{\left(i+1\right)}$ represents the overall degree of similarity (for all frequency points) between the current local surrogate model and its corresponding coarse model. If $\delta_{r}^{\left(i+1\right)}\geq \delta_{1}$ (here, $\delta_{1}$=0.7), it means the current surrogate model is accurate, and the next local region size can be expanded ($d^{\left(i+1,j\right)}=2d^{\left(i,j\right)}$). If $\delta_{r}^{\left(i+1\right)}\leq \delta_{2}$(here, $\delta_{2}$ = 0.3), it indicates the current surrogate may not be accurate enough to reflect the true response of the current coarse model. Therefore, the next local region size is reduced ($d^{\left(i+1,j\right)}=d^{\left(i,j\right)}/2$). When $\delta_{2}\leq \delta_{r}^{\left(i+1\right)}\leq \delta_{1}$, the next region size is kept the same as the current one and the starting point of the (*i*+1)th iteration is taken form the local optimal design found by the *i*th iteration.

**Figure 1:**
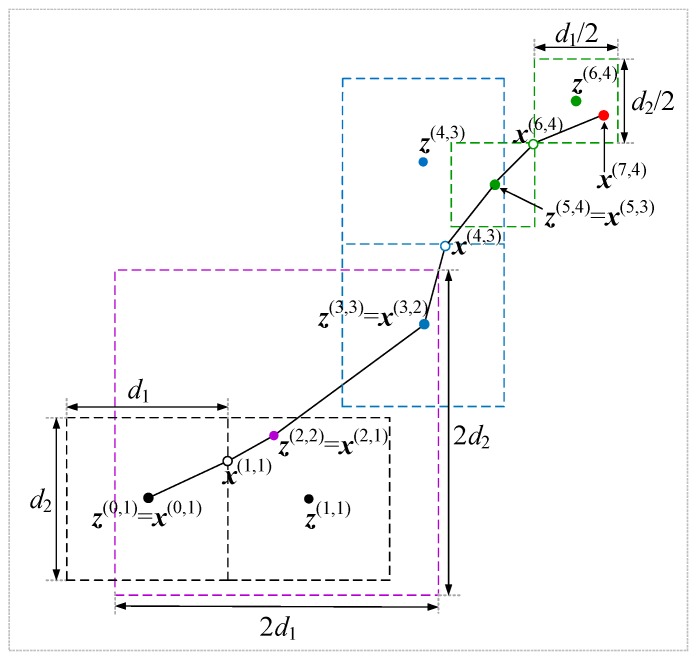
Multi-fidelity local surrogate model optimization (conceptual illustration for design parameter dimension *n* = 2). A local surrogate model $s^{\left(0,1\right)}$ (3) is established using $R_{c.1}$ in the initial region of size $\left[d_{1}\;d_{2}\right]$ (corresponding to $d^{\left(0,1\right)}={\left[d_{1}/2\;d_{2}/2\right]}^{T}$). It is optimized to yield a new design $x^{\left(1,1\right)}$, where the next surrogate model $s^{\left(1,1\right)}$ is established and centered at $z^{\left(1,1\right)}$. After $z^{\left(2,2\right)}=x^{\left(2,1\right)}$ is found, the algorithm enters the second iteration and the next surrogate model is built on $R_{c.2}$ with an enlarged local region size assuming a judgement factor $\delta_{r}^{}>0.7$. The process continues and terminates after 4 iterations at $x_{s}^{*}=x^{\left(7,4\right)}$.

The multi-fidelity surrogate models are built on various fidelity of the coarse models. Each iteration in the algorithm corresponds to a coarse model $R_{c.j}$ at specific fidelity. The algorithm converges in *K* iterations and *K* coarse models of different fidelity are used. Figure 1 shows the conceptual illustration of the process. The process in the figure exploits four coarse models:$R_{c.1}$, $R_{c.2}$, $R_{c.3}$, and $R_{c.4}$. For each iteration a particular color is used to mark local regions, their centers, and local optimal designs. The dashed line is used to mark the local regions in which the surrogate model is established. The solid dot $z^{\left(i,j\right)}$ is the center of the local region and the hollow circles is the current local optimal design $x^{\left(i,j\right)}$. The red dot represents the optimal design of the surrogate model obtain after *K* = 4 iterations.

### 2.4. Refinement of Surrogate with Fine Model

The optimized design $x_{s}^{*}$ of the last local surrogate model based on $R_{c.K}$ (closest to the fine model accuracy) is a good estimation of the optimal design of the fine (high-fidelity) model. To improve the accuracy of the optimal design for the fine model, the last surrogate model is further refined. The refinement process is performed in the local region centered at $x_{s}^{*}$. The surrogate model $s^{*}(\cdot )$ is established using coarse model $R_{c.K}$ within this region following (3) and (4).

In order to improve the surrogate model $s^{*}(\cdot )$, an input space mapping method [14] is applied. A vector $c^{*}$ is obtained to improve the matching between $s^{*}$ and fine model $R_{f}$ in the parameter extraction process

(7) $$c^{*}=\arg\underset{c}{\min}\left\|R_{f}(x_{s}^{*})-s^{*}(x_{s}^{*}+c)\right\|.$$

Then an output space mapping [15] correction term $R_{f}(x_{s}^{*})-s^{*}(x_{s}^{*}+c^{*})$ is added to the surrogate model to reduce the zeroth-order error [16]. The refined optimal design is then found as

(8) $$x^{*}=\arg\underset{x_{s}^{*}-d\leq x\leq x_{s}^{*}+d}{\min}U\left(s^{*}(x+c^{*})+[R_{f}(x_{s}^{*})-s^{*}(x_{s}^{*}+c^{*})]\right),$$

where d is the same as the last local region in Section 2.3. The refinement for local surrogate requires only one evaluation of $R_{f}$.

### 2.5. Summary of Multi-Fidelity Local Surrogate Model Optimization

**Figure 2:**
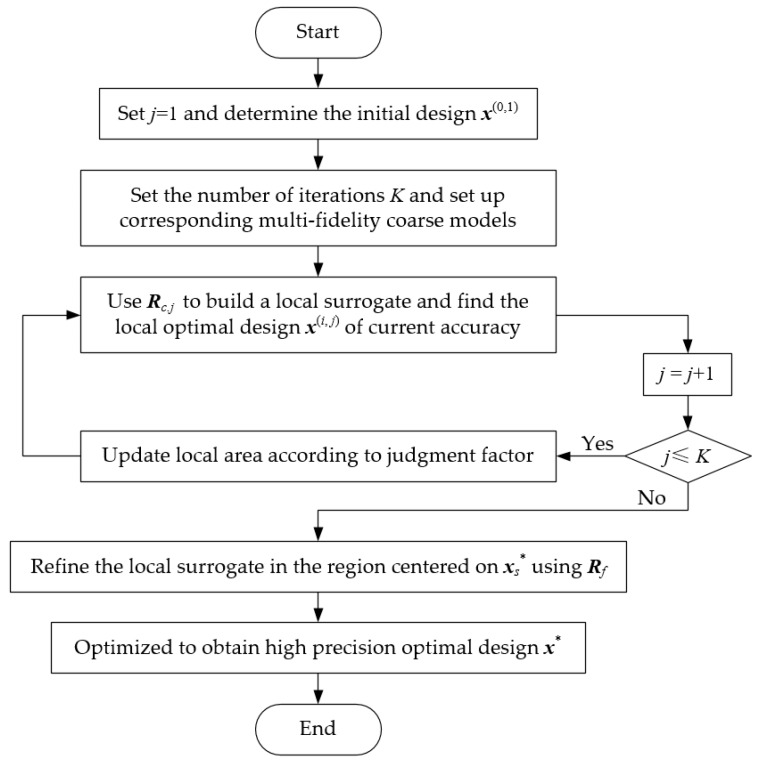
Complete flowchart of the proposed algorithm.

Based on the above description, Figure 2 summarizes the complete flowchart of the proposed algorithm.

## 3. Verification Examples and Comparison

In this section, we present the numerical verifications of the multi-fidelity local surrogate model optimization algorithm in Section 2. Our proposed algorithm is applied to design EM-based microwave component models. A microstrip filter, an UWB monopole antenna, and a compact UWB MIMO antenna are considered. We compare the performance of the proposed method with that of the original algorithm [12] and direct fine model optimization (genetic algorithm). To make a fair comparison, we used a moderate mesh size for $R_{c}$ when testing the original algorithm. For example, the second coarse model $R_{c.2}$ of the proposed algorithm is used as $R_{c}$ for the original algorithm in our comparison. To determine the mesh size of the coarse model $R_{c.j}$ in the proposed algorithm, we performed a preliminary test. It is found that when the mesh size of the coarse model is set so that the number of cells per wavelength are less than 10, the simulation response of the coarse model cannot represent the response characteristics of a fine model, therefore cannot be used. Generally, three to five iterations (or number of coarse models) are needed in the proposed algorithm. The mesh sizes of coarse models for $R_{c.1}$, $R_{c.2}$, and $R_{c.3}$ are set to 10, 15, and 20 cells per wavelength respectively in our examples.

### 3.1. A Microstrip Filter Optimization

**Figure 3:**
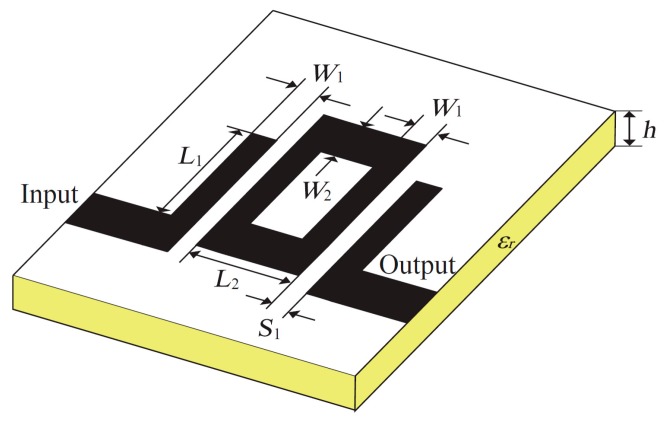
Second-order ring resonator bandpass microstrip filter: geometry [12,17].

The first example is a second-order ring resonator microstrip bandpass filter, which is proposed in [17]. The geometry of the microstrip filter is shown in Figure 3. There are five designable parameters: ***x*** = [*L*_1_, *L*_2_, *S*_1_, *W*_1_, *W*_2_]*^T^* mm. The initial design is $x^{\left(0,1\right)}={\left[18,\;24,\;0.3,\;1,\;1.3\right]}^{T}$ mm. FR-4 (loss free) is selected as the substrate ($\varepsilon_{r}=4.3$) and substrate height is *h* = 1.52 mm.

We use the vector $d^{\left(0,1\right)}={\left[1.0,\;1.0,\;0.1,\;0.1,\;0.1\right]}^{T}$ mm to define the size of the initial local region. The design specifications of the filter are $\left|S_{21}\right|\geq -1\;\mathrm{dB}$ (1.9 GHz≤ω≤2.1 GHz) and $\left|S_{21}\right|\leq -20\;\mathrm{dB}$ (1.0 GHz≤ω≤1.7 GHz and 2.3 GHz≤ω≤3.0 GHz). The design problem is translated into the optimization problem as follows

(9) $$\begin{array}{l} x=\arg\underset{x}{\min}(w_{1}\sum_{1.0\leq \omega \leq 1.7}\max\left(\left(R_{f}(x,\omega )+20\right),0\right)+w_{2}\sum_{1.9\leq \omega \leq 2.1}\max\left(\left(-1-R_{f}(x,\omega )\right),0\right)\\ \;\;+w_{3}\sum_{2.3\leq \omega \leq 3.0}\max\left(\left(R_{f}(x,\omega )+20\right),0\right)) \end{array}$$

where *w*_1_, *w*_2_, and *w*_3_ are weighting factors for different frequency bands (here, $w_{1}=1,w_{2}=10,$ and $w_{3}=5$). And $R_{f}(x,\omega )$ is the fine model frequency response value at ω GHz.

The filter structure is simulated using Computer Simulation Technology (CST) Microwave Studio transient solver with Intel(R) Core(TM) i7-4790 processor and 16 GB of RAM. The fine model $R_{f}$ has 253,561 meshes and its evaluation time is 5 min. To optimize the filter using multi-fidelity local surrogate algorithm of Section 2, we use two low-fidelity models: $R_{c.1}$ (40,480 meshes at $x^{\left(0,1\right)}$, evaluation time 0.6 min) and $R_{c.2}$ (96,330 meshes, 1.3 min). As a comparison, the filter is also optimized using the original algorithm, in which the low-fidelity model $R_{c}$ is the same as $R_{c.2}$ with 96,330 meshes and evaluation time of 1.3 min.

**Figure 4:**
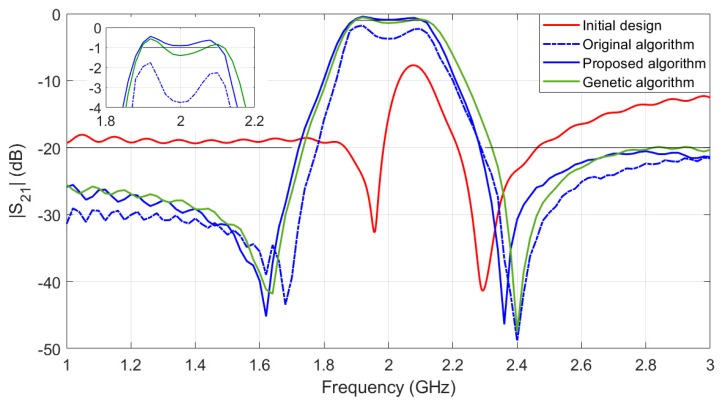
Second-order ring resonator bandpass microstrip filter: responses of the initial design and optimal designs obtained by various algorithms.

Figure 4 shows the responses of fine model $R_{f}$ at $x^{\left(0,1\right)}$ and at its optimal designs $x_{}{}^{*}={\left[19.81,\;23.48,\;0.32,\;0.88,\;1.48\right]}^{T}$ mm. In addition, the figure also compares the response of the optimal designs of the original algorithm (optimal design $x_{1}{}^{*}={\left[18.16,\;24.96,\;0.49,\;0.86,\;1.47\right]}^{T}$ mm) and direct optimization algorithm (using genetic algorithm and optimal design $x_{2}{}^{*}={\left[19.26,\;23.64,\;0.33,\;0.87,\;1.51\right]}^{T}$ mm).

The optimization costs of the three algorithms are compared in Table 1. The original algorithm terminated after 5 local surrogates’ optimization without finding the design satisfying the specifications. The direct optimization (genetic algorithm) obtains a design almost satisfying the specification, but it consumes a lot of computational cost (397 fine model evaluations). Our proposed algorithm obtains a design satisfying the specification and the total optimization cost is equivalent to only about 16 fine model $R_{f}$ evaluations. The computational saving of the proposed method is 95% or even more as compared to the direct optimization of the fine model.

### 3.2. Compact UWB MIMO Antenna Design

The second example is to design a compact UWB MIMO antenna using the proposed algorithm. In the design process, two optimization steps. In the first step, a compact UWB monopole antenna is optimized using our proposed method. In the second step, a MIMO antenna (constructed using two optimized UWB monopole antennas) is optimized using a simplify version of our proposed method.

#### 3.2.1. UWB Monopole Antenna Optimization

**Figure 5:**
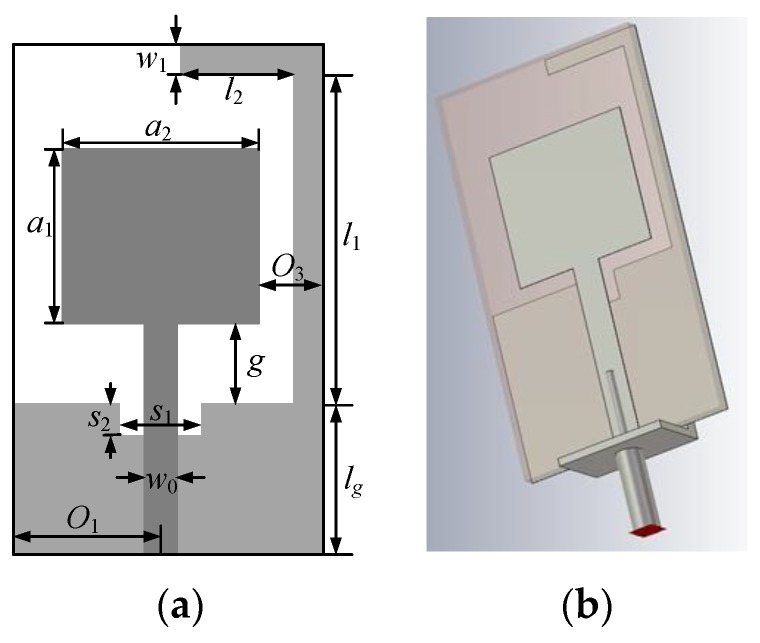
The UWB monopole antenna [19]: (**a**) Geometrical details of the radiator and ground plane; (**b**) 3D visualization of the antenna with the SMA connector in CST (The design is electrically small. In order to ensure the reliability of the simulation results, the electromagnetic (EM) model is simulated with an SMA connector attached).

First, the monopole antenna structure shown in Figure 5 is optimized. The structure consists of a rectangular radiating plate fed through a microstrip line and a ground plane with a small rectangular slot and a long ground stub. According to Reference [18], the small rectangular slot with a size of $s_{1}\times s_{2}$ is for better impedance matching at high frequencies and the long stub for isolation enhancement is bent to reduce the overall antenna area. It is designed on a Rogers substrate, RO4350B ($\varepsilon_{r}=3.55,$ tanδ=0.0037, and h=0.8 mm).

In this case, 11 design variables $x={\left[l_{g},\;g,\;a_{1},\;a_{2},\;l_{1},\;l_{2},\;w_{1},\;s_{1},\;s_{2},\;o_{1},\;o_{3}\right]}^{T}$ mm are considered. The feedline width is fixed at $w_{0}=1.8$ mm. The initial design is $x^{\left(0,1\right)}={\left[7,\;1,\;10,\;10,\;11,\;3,\;1,\;3,\;1,\;6,\;1\right]}^{T}$ mm. The size of the initial local region is defined as $d^{\left(0,1\right)}={[1,\;1,\;0.1,\;0.1,\;1,\;1,\;0.1,\;1,\;0.1,\;1,\;0.1]}^{T}$ mm. The fine model $R_{f}$ is evaluated using the CST (1168614 mesh cells, evaluation time is 6.5 min). We optimized this example using three low-fidelity models: $R_{c.1}$ (134560 meshes, 1 min), $R_{c.2}$ (187110 meshes, 1.5 min), and $R_{c.3}$ (338680 meshes, 2.8 min). As a comparison, this example is also optimized using the original algorithm, in which the low-fidelity model $R_{c}$ is the same as $R_{c.2}$ with 187110 meshes and evaluation time of 1.5 min.

The optimization goal is to meet the following specifications: reflection coefficients $\left|S_{11}\right|\leq -12$ dB for 3.1 GHz to 10.6 GHz. In addition, the antenna footprint $S(x)=(o_{1}+0.5\cdot a_{2}+o_{3})\times (l_{g}+l_{1}+w_{1})$ is also considered while maintaining the above electrical performance. The size reduction is selected as the main optimization problem, and good electrical performance is achieved in the form of a penalty function. Thus, the local region optimization according to the formula (2) is defined as

(10) $$x_{}^{\left(i,j\right)}=\arg\min\left(S(x)+\beta \cdot c\left(s_{}^{\left(i,j\right)}(x)\right)\right),$$

where β is a penalty factor (here, β=1000) and $s_{}^{\left(i,j\right)}(\cdot )$ is the *i*th multi-fidelity surrogate model built using $R_{c.j}$, (*j* = 1, 2, 3). And c is a penalty function defined as

(11) $$c(s^{\left(i,j\right)}(x))=\max\left\{\max\left\{{\left|S_{11}\right|}_{3.1\mathrm{GHz}\;\mathrm{to}\;10.6\mathrm{GHz}}\right\}+12,0\right\}.$$

**Figure 6:**
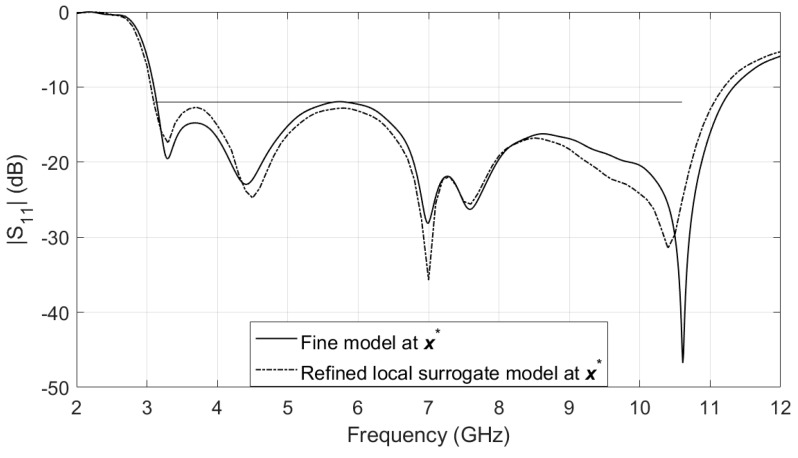
A UWB monopole antenna: response of the optimal design in the refined local surrogate model and in the fine model.

After the optimal solution of the final local surrogate model is found, the surrogate model is refined. The optimal design $x^{*}={\left[11.2,\;0.7,\;8.85,\;8.77,\;13.8,\;5.06,\;1.01,\;2.3,\;1.9,\;6.96,\;1.71\right]}^{T}$ obtained by the refined local surrogate model (8) is very close to that of the fine model and they both satisfy the specification, as shown in Figure 6.

**Figure 7:**
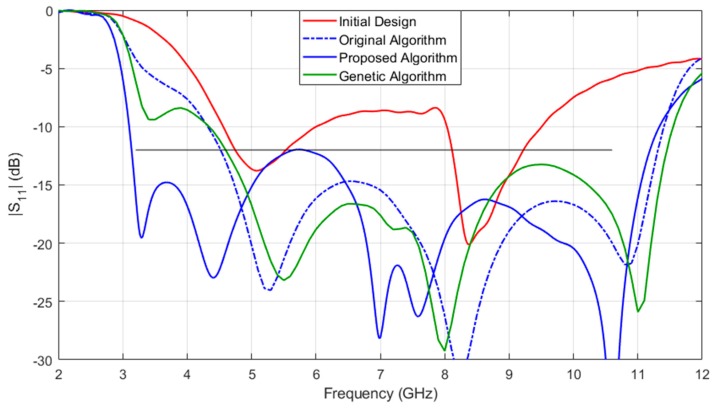
A UWB monopole antenna: responses of the initial design and optimal designs obtained by various algorithms.

Figure 7 shows the response of fine model $R_{f}$ at initial design $x^{\left(0,1\right)}$ and the optimal design obtained x^*^ by the proposed algorithm. The corresponding antenna size is 13 mm × 26 mm (338 mm^2^). In addition, the figure also shows results obtained by the original algorithm and the direct optimization algorithm (genetic algorithm). It can be seen that the optimal design $x_{1}^{*}={\left[8.96,\;0.64,\;7.78,\;8.46,\;13.16,\;3.7,\;0.97,\;2.18,\;2.07,\;6.7,\;1.8\right]}^{T}$ of original algorithm does not satisfy the design specification. One of the optimization results of genetic algorithm $x_{2}^{*}={\left[10.84,\;0.64,\;9.13,\;8.68,\;14.04,\;4.82,\;1.14,\;2.13,\;1.84,\;7.12,\;1.59\right]}^{T}$ is also shown in Figure 6 (three runs of genetic algorithm are performed and none of the results satisfies the design specification).

The optimization costs of UWB monopole antenna using the three algorithms are compared in Table 2. The original algorithm terminated after 4.2 h without finding the design satisfying the specifications. The direct optimization (genetic algorithm) is not able to obtain a design satisfying the specification after an average of 53.8 h (CST genetic algorithm terminates after 497 fine model evaluations by default). Our proposed algorithm obtains a design satisfying the specification and the total optimization cost is 5.6 h.

#### 3.2.2. Compact UWB MIMO Antenna

**Figure 8:**
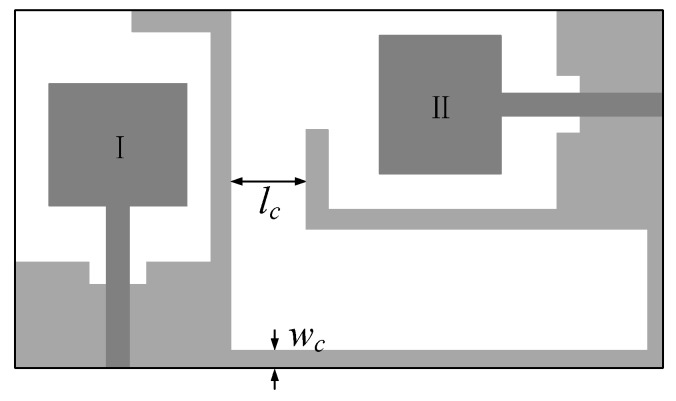
Compact UWB MIMO antenna: geometry [19].

We construct a UWB MIMO antenna using two radiating patches (UWB monopole antenna element) placed perpendicularly. Figure 8 shows the geometry of the compact UWB MIMO antenna. A thin ground strip is used to electrically connect the two ground planes together. The ground strip width and length affect lower cutoff frequency and the isolation.

The optimal design x^*^ in the Section 3.2.1 is used as the geometric parameter of the two UWB monopole antenna elements in the MIMO antenna. At this time, the design variable $x={\left[l_{c}\;w_{c}\right]}^{T}$ of the MIMO antenna are optimized.

**Figure 9:**
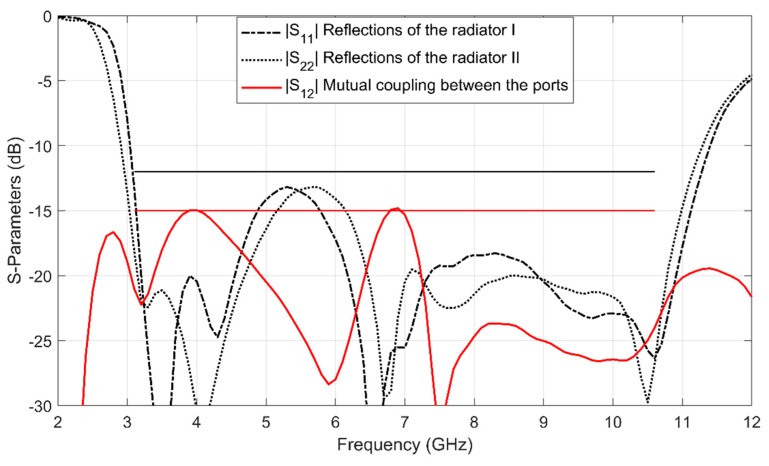
S-parameter characteristics of the compact UWB MIMO antenna: responses of the optimal design $x^{*}$. The black solid straight line is the specification (−12 dB) for reflection of each radiator. The red solid straight line is the specification (−15 dB) for mutual coupling.

The MIMO antenna is complex and only the high-fidelity EM simulation is reliable. The number of design variables is only two. For these reasons, a simplified version of our multi-fidelity local surrogate model optimization algorithm is used. Instead of multiple coarse models, the algorithm directly use the response of the fine model to build the local surrogate models, $R_{c.j}=R_{f},\;j=1,\ldots ,K$. The initial design is $x^{\left(0,1\right)}={\left[3,\;3\right]}^{T}$ mm. The specifications are mutual coupling $\left|S_{21}\right|\leq -15\;\mathrm{dB}$ for 3.1 GHz to 10.6 GHz. The optimal design $x^{*}={\left[4.68\;2.21\right]}^{T}$ mm is obtained by establishing and optimizing three local surrogate models iteratively. Figure 9 shows the response of $R_{f}$ at the optimal design $x^{*}$. As can be seen, the return loss $\left|S_{11}\right|$ (or $\left|S_{22}\right|$) of the MIMO antenna is less than −12 dB for 3.1 GHz to 10.6 GHz. Since the geometry parameter values of the UWB monopole antenna elements are the same, the return loss response curves at the two ports are similar. The mutual coupling of less than −15 dB between the input ports is achieve (the compact UWB MIMO antenna obtained high isolation).

## 4. Conclusions

A novel multi-fidelity local surrogate model optimization approach for microwave component design is presented. In this optimization process, multiple local surrogate models are established and optimized iteratively. It exploits a series of coarse models with variable fidelity to construct the local surrogate models with sequentially increased accuracy. The proposed algorithm introduces the judgment factor to implement adaptive local surrogate model optimization. The algorithm converges quickly and efficiently to the optimal design of fine model. This is demonstrated through a microstrip filter and a compact UWB MIMO antenna design examples Compared with the original local surrogate model optimization algorithm, the accuracy of the optimal design is improved when applying the proposed algorithm. Compared with the direct optimization algorithm, the proposed algorithm finds the optimal design efficiently.

## Figures and Tables

**Table 1:** Second-order ring resonator bandpass microstrip filter: optimization cost.

Method	Required Model Evaluation	Number of Model Evaluations	Optimization Cost	
			Absolute (min)	Relative to $R_{f}$
Direct optimization	Fine model $R_{f}$	397	1985	397
Original algorithm	$\mathrm{Coarse}\;\mathrm{model}\;R_{c}$ $\mathrm{Fine}\;\mathrm{model}\;R_{f}$ Total optimization time	55 2 N/A	71.5 10 81.5	14.3 2 16.3
Proposed algorithm	$\mathrm{Coarse}\;\mathrm{model}\;R_{c.1}$ $\mathrm{Coarse}\;\mathrm{model}\;R_{c.2}$ $\mathrm{Fine}\;\mathrm{model}\;R_{f}$ Total optimization time	22 44 2 N/A	13.2 57.2 10 80.4	2.6 11.4 2 16

**Table 2:** A UWB monopole antenna: optimization cost.

Method	Required Model Evaluation	Number of Model Evaluations	Optimization Cost	
			Absolute (h)	Relative to $R_{f}$
Direct optimization	$\mathrm{Fine}\;\mathrm{model}\;R_{f}$	497	53.8	497
Original algorithm	$\mathrm{Coarse}\;\mathrm{model}\;R_{c}$ $\mathrm{Fine}\;\mathrm{model}\;R_{f}$ Total optimization time	161 2 N/A	4 0.2 4.2	38 2 40
Proposed algorithm	$\mathrm{Coarse}\;\mathrm{model}\;R_{c.1}$ $\mathrm{Coarse}\;\mathrm{model}\;R_{c.2}$ $\mathrm{Coarse}\;\mathrm{model}\;R_{c.3}$ $\mathrm{Fine}\;\mathrm{model}\;R_{f}$ Total optimization time	69 46 69 2 N/A	1.1 1.1 3.2 0.2 5.6	10 10 30 2 52
